# Deposit Limit Prompt in Online Gambling for Reducing Gambling Intensity: A Randomized Controlled Trial

**DOI:** 10.3389/fpsyg.2019.00639

**Published:** 2019-03-28

**Authors:** Ekaterina Ivanova, Kristoffer Magnusson, Per Carlbring

**Affiliations:** ^1^Division of Clinical Psychology, Department of Psychology, Stockholm University, Stockholm, Sweden; ^2^Centre for Psychiatry Research, Department of Clinical Neuroscience, Karolinska Institutet, Stockholm, Sweden; ^3^Stockholm Health Care Services, Stockholm County Council, Stockholm, Sweden; ^4^Department of Psychology, University of Southern Denmark, Odense, Denmark

**Keywords:** problem gambling, online gambling, responsible gambling, deposit limit, pre-commitment, natural gambling environment, consumer protection

## Abstract

Pre-commitment tools – allowing users of gambling services to pre-set a limit for how much money they may spend – are relatively common. However, there exist no clear evidence of their effectiveness in preventing gamblers from spending more money than they otherwise planned. The aim of the study was to compare gambling intensity between users of an online gambling service prompted to set a deposit limit and non-prompted customers, both in the whole sample and among most active users based on the total number of gambling days. Prospective customers of a publicly governed gambling operator from Finland were randomized to receive a prompt to set a voluntary deposit limit of optional size either (1) at registration, (2) before or (3) after their first deposit, or (4) to an unprompted control condition. Data on customers from Finland with online slots as a preferred gambling category (*N* = 4328) were tracked in the platform for 90 days starting at account registration, gambling intensity being measured with aggregated net loss. The intervention groups did not differ from each other in either proportion of participants with positive net loss or size of positive net loss. The pooled intervention group did not differ from the control group regarding proportion of gamblers with positive net loss (OR = 1.0; *p* = 0.921) or size of net loss (*B* = -0.1; *p* = 0.291). The intervention groups had higher rates of limit-setters compared to the control condition (OR_at-registration/pre-deposit/post-deposit_ = 11.9/9.2/4.1). Customers who have increased/removed a previously set deposit limit had higher net loss than the limit-setters who have not increased/removed their limit (B_at-registration/pre-deposit/post-deposit/control_ = 0.7/0.6/1.0/1.3), and unprompted limit-setters lost more than unprompted non-setters (*B* = 1.0). Prompting online gamblers to set a voluntary deposit limit of optional size did not affect subsequent net loss compared to unprompted customers, motivating design and evaluation of alternative pre-commitment tools. Setting a deposit limit without a prompt or increasing/removing a previously set limit may be a marker of gambling problems and may be used to identify customers in need of help.

## Background

Problem gambling, understood as experiencing negative consequences of using gambling services (Cowlishaw and Kessler, 2016), is prevalent in 0.12–5.8% of the population in all the parts of the world (Calado and Griffiths, 2016), and is recognized as a public health issue in many countries (Adams et al., 2009; Marshall, 2009; Svensson et al., 2013; Gainsbury et al., 2014). With the increase in the number of Internet gambling services (Scholes-Balog and Hemphill, 2012) concerns have been raised about potential harms related to its specific features, such as high accessibility, absence of social control, and high speed (Gainsbury et al., 2015). Consequently, the need to develop adequate protective measures has been addressed (Gainsbury et al., 2013). The particular popularity of slot games had been stressed when speaking of land-based gambling (Chen et al., 2013), and it is suggested that slot games do stand out among other gambling forms with regard to gambler characteristics as well as its representativeness among problem gamblers (Clarke, 2005; Dowling et al., 2005; Balodis et al., 2014; Binde et al., 2017). In online environments, playing slot games seems to be associated with elevated rates of gambling problems (McCormack et al., 2013).

The capacity of online gambling platforms to track customers’ activity (Coussement and De Bock, 2013; Martin, 2016) has been emphasized as a potential tool for creating safer gambling environments (Griffiths et al., 2009). Responsible gambling (RG) tools - defined as features aiming to help individuals control their gambling behaviors (Blaszczynski et al., 2004, 2011; Echeburua and De Corral, 2008) and addressed by policy makers and industries (Hing and Mackellar, 2004; Wood et al., 2014; O’Mahony and Ohtsuka, 2015) - are now being developed in online gambling settings (Forsström et al., 2016) focusing on behavioral feedback (Auer et al., 2014; Auer and Griffiths, 2015a,b, 2016; Wood and Wohl, 2015), self-exclusion (Griffiths et al., 2009), and predicting gambling problems (Adami et al., 2013; Dragičevic et al., 2015; Luquiens et al., 2016; Percy et al., 2016).

One RG-strategy that has the potential to help individuals gamble in a sustainable manner, is setting pre-committed limits for how much money they can lose, deposit or win (Auer and Griffiths, 2013; Kim et al., 2014; Walker et al., 2015). However, three systematic reviews failed to find clear evidence of the effectiveness of monetary pre-commitment (Ladouceur et al., 2012, 2017; Drawson et al., 2017). Online gamblers find voluntary limits useful, whereas mandatory limits are viewed as patronizing (Bernhard et al., 2006; Griffiths et al., 2009; Gainsbury et al., 2013). However, Nelson and colleagues (Nelson et al., 2008) observed that only 1.2% of users of an online betting website (*N* = 567 out of *N* = 47,134) used the available deposit-limit feature, possibly indicating differences between the populations of gamblers as well as discrepancy between attitude and behavior. Positive attitudes might be insufficient to facilitate the use of the tool. Also, an active setting of limits is not necessary for those who already limit their gambling through minimal play, for instance by only depositing a sum they are willing to spend and not going beyond it. The self-limiters showed higher gambling intensity than the rest of the sample, and the intensity was reduced slightly after setting the limit (Broda et al., 2008). Current evidence also suggests that problem gamblers are more likely to exceed self-imposed gambling limits compared to regular gamblers (Hing et al., 2015).

Prior research has underscored the importance of making expenditure decisions in a neutral versus aroused emotional state (Wilkes et al., 2010; Ladouceur et al., 2012), explaining potential effectiveness of pre-commitment tools. High levels of impulsivity associated with gambling problems (Bagby et al., 2007), lack of the ability to self-regulate specifically in the context of an attempted behavior change (Ricketts and Macaskill, 2003), and dissociating (Wanner et al., 2006) can make it difficult to stop depositing/betting while actively gambling. From the point of view of learning theory, making an expenditure decision prior to engaging in gambling activity would make sense due to the absence of establishing operations and reinforcers that otherwise occur during a gambling session and distort decision-making (Weatherly and Flanery, 2008; James and Tunney, 2016).

While the potential effectiveness of pre-commitment may seem logical, the results of existing trials are very mixed. Despite that, setting limits does occur as official recommendations from authorities (Australian Government, Productivity Commission, 2010; Williams et al., 2012) and is proposed as a mandatory requirement to the gambling industry (Statens Offentliga Utredningar [SOU], 2017). Apart from not being evidence-based, these recommendations could be used to unjustifiably market gambling services as being responsible, possibly inducing an ungrounded feeling of safety in gamblers and encouraging increased involvement in gambling without matching it with adequate protective measures. The current trial aims to [1] compare gambling intensity between customers with online slots as preferred gambling activity prompted to set a voluntary removable deposit limit of optional size with unprompted customers, to test the effect of the prompt on subsequent gambling intensity both regardless of gambling involvement and [2] in the subgroup of most involved gamblers. Online gamblers who are prompted to set a deposit limit are expected to exhibit a lower gambling intensity compared to unprompted gamblers. The trial also aims to [3] compare gambling intensity between three different intervention groups to study whether the point in time when the gamblers receive the prompt affects gambling intensity.

## Materials and Methods

### Trial Design

**FIGURE 1:**
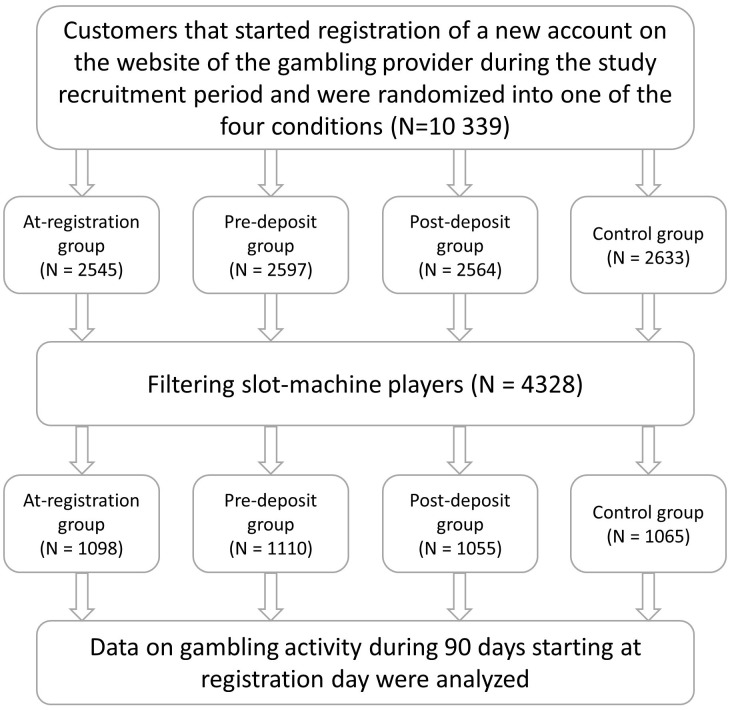
Recruitment and intervention flowchart.

The data was collected in the online platform of a publicly governed gambling company from the Åland Islands (an autonomous region in Finland), running an online gambling service and providing slot-games, poker, betting, casino games, and bingo. Bonuses and campaigns were in place when the trial was conducted, but no loyalty schemes were implemented. The most common slot games available are the ones resulting in a win or no-win in each spin and the spins are independent from each other. Other common slot types are the ones accumulating points in games with low return to player in order to come to a bonus game with high return to player and the ones where the bet level depends on the outcome of the previous spin. Starting in 2016, all prospective customers who registered an account on the website were randomized to either be prompted to set a deposit limit (1) during the registration process (at-registration group), (2) before they were about to make their first deposit (pre-deposit group), or (3) right after they made their first deposit (post-deposit group), or (4) to a control condition (no prompt). The randomization was performed independently of the authors in the platform using *java.security.SecureRandom* - a component of the Java programming language. The research group received access to the data on the gambling activity of 10 339 randomized customers during a 90-day period following the users’ registration. Among the initial sample, online slot players were identified, resulting in the final sample of 4328 customers (see Figure 1). The study was approved by the Regional Ethics Committee in Stockholm, Sweden (registration number 2016/1924-31). When registering an account on the gambling website, all prospective customers actively consent to the data being used for research purposes. No additional written consent was necessary (which was explicitly approved by the Regional Ethics Committee in Stockholm, see above) due to the non-invasive character of the intervention (a one-time prompt to set a voluntary deposit limit of optional size) and the fact that no sensitive personal data were collected. The hypotheses and the proposed analysis plan were pre-registered at Open Science Framework (osf.io^1^). The post-registration decision to focus on slot-players was taken in order to increase the generalizability of the results. It was assumed that a sample of online slot players would be more representative for different populations of online slot players than a sample of mixed players in relation to different populations of mixed players, as higher heterogeneity is expected among the mixed populations. Focusing on slot players is also justified with regard to its distinct position among the common gambling types (Clarke, 2005; Dowling et al., 2005; Chen et al., 2013; Balodis et al., 2014; Binde et al., 2017).

### Participants

The customers included in the initial data-set were playing in Finland and were at least 18 years of age according to the personal identification number required at registration. Data on customers with online slots as the preferred gambling category for more than 80% of their total gambling days was analyzed. A gambling category was identified as preferred during a day when the customer wagered more money on said category than on any other category. No lower limit for gambling activity was required, meaning playing a single slot game was enough to be identified as a slot-machine player, as long as no other games were played.

### Interventions

The prospective customers were randomized into one of the conditions when they started registering an account on the website. If a prospective customer canceled the registration and started over at a later stage, they kept the earlier randomization if they used the same device and if the cache on their device was not cleared.

The customers in the control condition were not prompted with the possibility of setting a deposit limit but had access to the option under the “Safe gaming tools” tab on their profile page. There was a possibility of setting a weekly and a monthly limit that would be in force until the customer actively removed it. A decreased limit was applied immediately, and an increased or removed limit was applied seven days after the change. When the limit was reached, the new deposits were declined with the message: “The deposit exceeds your [weekly/monthly] limit. Click here to change the limit.” until the period of the limit was over. The deposit limits were not slot-specific but applied to all gambling activity in the platform.

The customers in the other three conditions were exposed to the possibility of setting a deposit limit. The at-registration group was prompted to set a deposit limit while filling in the registration form; they saw the message: “Smart players keep track of their spending. How much are you prepared to spend?” This was followed by a registration bar in which customers could enter a weekly deposit amount, and they could select a checkbox marked: “I want to choose it later.” To communicate the nature of the limit, a default text “Weekly deposit limit” was shown in the registration bar before the customer entered their own value. The pre-deposit group was shown a window with the message, “Choose how much you want to spend,” triggered by the customer trying to make a first deposit. The content of the window was identical to the offer in the at-registration group, but the customers could also decline the offer by clicking the cross-button. The post-deposit group was subjected to the same procedure as the pre-deposit group, only after the customers made their first deposit. If a customer in an intervention group declined the offer, they could set a deposit limit by opening “Safe gaming tools”- tab.

### Outcome Measures

Gambling intensity was measured using customers’ aggregated net loss (NL) as primary outcome measure, calculated as the total sum of wagers and winnings during the 90-day period after registration. Initially, theoretical loss (TL) was defined as the study’s primary outcome measure. TL is defined as the customer’s real money stakes multiplied by the proportion of the stake customers are expected to lose if they play the game in an optimal way an infinite number of times. However, the research team gained additional insights about the limitations of using TL in the sample of the current size and with the relatively low gambling involvement in a large proportion of the sample. Moreover, researchers have previously expressed concerns about suitability of TL as a measure of gambling intensity (Tom and Shaffer, 2016). TL was abandoned as primary outcome measure as it was considered a less stable indicator of gambling intensity compared to NL in the current sample. Inclination to set and increase or remove a deposit limit, as well as total sum of deposits and total number of active gambling days during the period of data collection were used as secondary outcome measures. A gambling day was defined as a day when a customer placed at least one bet without subsequently canceling it. The totals of NL, deposits and number of gambling days were analyzed across the platform and were not slot specific.

The data were delivered to the research team as anonymized spreadsheets with a unique identification number assigned to each included customer on the April 21, 2017. The customers’ gender (male/female) was self-provided during the registration, the age was calculated by the gambling operator during data extraction using the personal identification number and the remaining measures were tracked by the platform.

### Statistical Analyses

The analyses were carried out using the statistical software R version 3.5.0 (R Core Team, 2018). Player NL and deposits in euros were aggregated per individual over the 90-days data collection period. Prior to carrying out the analyses, the NL and deposit values were adjusted to make the whole sample medians equal 100 due to corporative financial confidentiality issue ([adjusted value] = [real value in euros] × 100/[median of real value in euros]). The transformation is linear and does not affect between-group comparisons. The inclination to set a deposit limit was analyzed using a logistic regression model. The differences in NL between the customers who chose not to set, set or increase their deposit limit were studied using a logistic regression model (with the binary variable of positive or non-positive aggregated NL) and using linear regression on log-transformed NL for the customers with positive aggregated NL.

Randomization groups were compared regarding aggregated NL, sum of deposits and total number of gambling days. NL could take both positive and negative values depending on whether the customer lost or won money and positive NL means the customer lost money during data collection period, sum of stakes is higher than sum of winnings. Positive NL, sum of deposits and total number of gambling days were log-transformed prior to conducting the analysis. Between-group differences in NL were also conducted on the subgroup of 10% of most intensive gamblers based on the total number of active gambling days. Bayes factors of alternative hypothesis over null hypothesis (BF_10_) were calculated for the differences between the three intervention groups regarding proportion of individuals with positive NL (whole sample: BF_10_ = 0.007, most involved subgroup: BF_10_ = 0.030), size of NL among individuals with positive NL (whole sample: BF_10_ = 0.016, most involved subgroup: BF_10_ = 0.082), sum of deposits (BF_10_ = 0.002) and total number of gambling days (BF_10_ = 0.001). As all BF_10_ were under 0.33 (Dienes, 2014), the three intervention groups were pooled to be compared to the control group. Between-group differences in proportion of individuals with positive NL were analyzed using general linear model. Differences in positive NL, sum of deposits and total number of gambling days were studied using linear regression (see Supplementary Figure 1 for model fit). Age and gender were added as independent variables in all linear models.

BF_10_ were calculated for the between-group comparisons in order to get a more nuanced picture of occasional differences and quantify the relative support for the null hypothesis over the alternative hypothesis. BF_10_ were calculated with the prior distribution set to Cauchy *r* = 0.5, and the values were interpreted as reported by Kass and Raftery (1995) with BF of 1–3.2 showing that the evidence for the alternative hypothesis is only worth a bare mention, BF 3.2 to 10 showing substantial evidence, BF 10 to 100 showing strong evidence, and BF >100 showing very strong evidence. Quantile regression with NL by randomization group was conducted over the entire distribution in order to identify occasional subgroups.

## Results

### Proportions of Limit-Setters

The proportion of males in the whole sample was 65.0% and there were no gender differences between the randomisation groups (Table 1, χ^2^(Gainsbury et al., 2014) = 2.39, *p* = 0.500). Mean age was 29.3 years (SD = 12.5) with no between group differences (Table 1, *F*(Gainsbury et al., 2014) = 0.174, *p* = 0.914). The proportion of limit setters was higher in all intervention groups compared to the control group (Table 1) and men were more likely to set a deposit limit (OR(95% CI) = 1.246 (1.069 – 1.451), *p* = 0.005), with no significant effect of age (OR(95% CI) = 0.996 (0.990 – 1.002), *p* = 0.187). No association was found between the proportion of limit increasers among limit setters and randomization group, age or gender (Table 1). Out of 4328 individuals, only *N* = 74 (1.7%) chose to decrease their deposit limit at least once after setting it and without ever increasing or removing it (N_at-registration/pre-deposit/post-deposit/control_ = 22/27/17/8).

**Table 1:** Baseline characteristics, proportions of limit setters and increasers (among limit setters), and sizes of the limits based on randomization group, gender and age.

	Control	At-registration	Pre-deposit	Post-deposit
N	1065	1098	1110	1055
Age: M(SD)	29.4 (12.5)	29.4 (12.8)	29.0 (12.2)	29.3 (12.5)
% males	65.5	65.0	66.0	63.0
% Limit set	6.5	45.0	38.8	21.9
OR (95%CI)	-	11.883^∗∗∗^ (9.056–15.592)	9.182^∗∗∗^ (6.993–12.058)	4.076^∗∗∗^ (3.066–5.417)
% Limit increased^1^	40.6	31.4	29.2	39.0
OR (95%CI)	-	0.674 (0.402 – 1.130)	0.609 (0.361 – 1.029)	0.939 (0.542 – 1.626)
**Limit sizes in euros (Weekly/Monthly)**				
Lowest	10/10	5/10	5/10	10/10
Median	40/50	50/50	50/60	50/50
95th percentile	305/373	500/950	200/500	400/920


*Significance levels: ^∗^<0.05, ^∗∗^<0.01, ^∗∗∗^<0.001. OR – odds ratio of having set or increased/removed deposit-limit at least once during the data collection period in each intervention group compared to control group. (1) Proportion of limit-setters who chose to increase or remove their limit at least once during the data collection period. Lowest: the lowest deposit amount observed in the specific category. The 95th percentile is chosen instead of the highest value because some of the highest values were extreme and unlikely to reflect what the customer thought was a reasonable limit. They seemed to serve the purpose of an absence of a deposit limit.*

### Deposit Limit Status and Gambling Intensity

**FIGURE 2:**
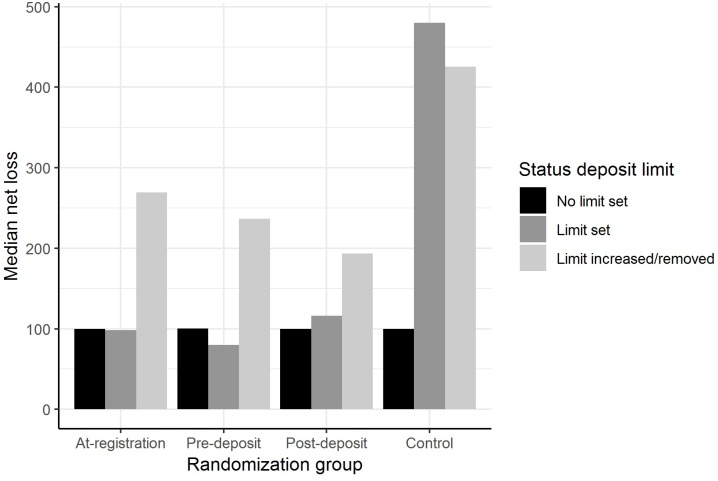
Median NL for customers who did not set a deposit limit, those who set a deposit limit (without removing it), and those who increased or removed a deposit limit. The numbers for the non-setters/setters-non-increasers/limit-increasers in the intervention groups are: at-registration group (604/339/155), pre-deposit group (679/305/126), post-deposit group (824/141/90), and control group (996/41/28).

Figure 2 shows median NL among individuals with different deposit limit status across the randomisation groups. The proportion of individuals with positive NL was higher among setters-non-increasers in pre-deposit group and lower among increasers in post-deposit group compared to the same proportion among non-setters in both cases (Table 2). Compared to non-setters, NL was higher among limit increasers across all randomisation groups and among setters-non-increasers in control group, as well as lower among setters-non-increasers in at-registration and pre-deposit groups (Table 2). Among limit-setters, 47% reached their limit (either weekly or monthly) at least once during the data collection period in at-registration group, the proportions were 53% in pre-deposit group, 61% in post-deposit group, and 74% in control group.

**Table 2:** NL differences between non-setters, setters-non-increasers and increasers in each randomization group.

	Control	At-registration	Pre-deposit	Post-deposit
N	1065	1098	1110	1055
% positive NL	79.2	79.1	80.5	77.5
Setters-non-increasers: OR(95% CI)	0.803 (0.388 – 1.666)	1.202 (0.859 – 1.680)	1.528^∗^ (1.059 – 2.204)	1.254 (0.794 – 1.979)
Increasers: OR(95% CI)	0.950 (0.380 – 2.374)	0.875 (0.576 – 1.327)	0.884 (0.562 – 1.393)	0.596^∗^(0.372 – 0.956)
Size of positive NL	*N* = 844	*N* = 868	*N* = 894	*N* = 818
Setters-non-increasers: B (95% CI)	1.035^∗∗∗^ (0.479 – 1.592)	-0.397^∗∗^ (-0.685 – -0.108)	-0.573^∗∗^ (-0.937 – -0.209)	-0.087 (-0.409 – 0.235)
Increasers: B (95% CI)	1.257^∗∗∗^ (0.601 – 3.753)	0.721^∗∗∗^ (0.329 – 1.113)	0.633^∗^ (0.102 – 1.164)	0.970^∗∗∗^ (0.544 – 1.396)


*NL – net loss, positive NL means the customer lost money during data collection period, sum of stakes is higher than sum of winnings. OR – odds ratio of having a positive NL compared to those not having set a deposit limit. B – estimated change in adjusted NL compared to those not having set a deposit limit. Significance levels: ^∗^<0.05, ^∗∗^<0.01, ^∗∗∗^<0.001.*

### Gambling Intensity and Between-Group Analyses

**FIGURE 3:**
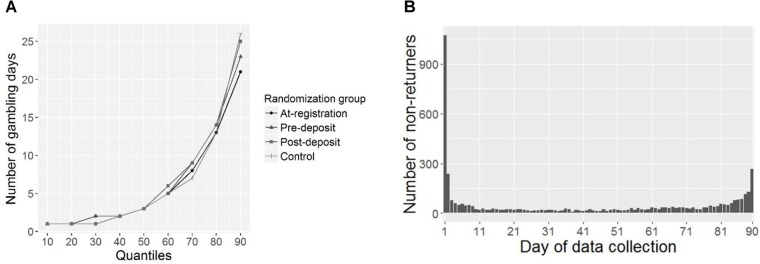
Quantile distribution of total number of gambling days in the randomization groups **(A)** and number of customers having their last gambling day for each day of data collection **(B)**. The highest 10th percentile of gamblers had between 26 and 90 gambling days in total. Out of the whole sample, 62.7% had at least 1 gambling day after the first 7 calendar days following the registration, the proportions are 50.7% after the first 30 calendar days and 37.6% after the first 60 calendar days. *N* = 1074 customers did not have an active gambling day after day number 1 of the 90-day data collection period, *N* = 237 did not return after day number 2, *N* = 25 did not return after day number 10, and *N* = 266 were active on day number 90.

Quantile distribution of number of gambling days for the whole sample and number of customers having their last gambling day for each day of data collection are shown in Figure 3. Most of the gambling activity occurred directly after the registration with more than 25% of customers not returning to the platform after the first gambling day. Median number of active gambling days across all randomization groups was three and only 10% of the analyzed individuals had more than 25 active gambling days. Median positive NL in the subgroup of the 10% most intensive players (based on total number of active gambling days) was around 20 times higher than that of the whole sample (Tables 3, 4). No effect of intervention group was found on either proportion of individuals with positive NL or the size of NL among the individuals with positive NL, neither in the whole sample (Table 3) nor in the 10% of most intensive gamblers in each randomization group based on total number of gambling days (Table 4). Higher age was associated with larger proportion of customers with positive NL and higher NL among those with positive NL both in the whole sample, and in the subgroup of the most involved customers. Being a male was associated with higher NL among the customers with positive NL in the whole sample. No effect of randomization group was found on the sum of deposits and total number of gambling days in the whole sample (Supplementary Table 1). Quantile regression showed lower levels of positive NL for the customers in the pre-registration group in higher quantiles and higher levels in post-registration group in lower quantiles (Supplementary Figure 2).

**Table 3:** Comparisons between the prompted groups and the control group regarding proportion of individuals with positive NL and size of NL among the individuals with positive NL.

	Control	At-registration	Pre-deposit	Post-deposit
Median NL	99.8	100.0	100.0	99.8
Proportion of individuals with positive NL	79.2 %	79.1 %	80.5 %	77.5 %
Between-group statistics	–	OR (95% CI) = 0.991 (0.836 – 1.176); *p* = 0.921; BF_10_ = 0.036		
Adjusted R^2^	0.003			

N with positive NL	*N* = 844	*N* = 868	*N* = 894	*N* = 818
Median NL	193	196	180	180
Between-group statistics	–	B (95% CI) = -0.080 (-0.229–0.069); *p* = 0.291; BF_10_ = 0.064		
Adjusted R^2^	0.045			


*NL – net loss, positive NL means the customer lost money during data collection period, sum of stakes is higher than sum of winnings. OR – odds ratio of having a positive net loss in the pooled prompted group compared to control group. B – estimated change in adjusted NL in the pooled prompted group compared to control group. BF_*10*_ – Bayes factor of the alternative hypothesis against the null hypothesis. For the proportion of customers with positive NL: estimates for effect of age (OR (95% CI) = 1.011(1.005–1.017), *p* < 0.001), estimates for effect of gender (being a male, OR (95% CI) = 1.112(0.950–1.300), *p* = 0.186). For the size of positive NL: estimates for effect of age (B (95% CI) = 0.034(0.029–0.039), *p* < 0.001), estimates for effect of gender (being a male, B (95% CI) = 0.149(0.012–0.287), *p* = 0.033).*

**Table 4:** For the 10% of most intensive gamblers based on the total number of gambling days: Comparisons between the prompted groups and the control group regarding proportion of individuals with positive NL and size of NL among the individuals with positive NL.

	Control	At-registration	Pre-deposit	Post-deposit
N	103	107	110	101
Median N gambling days	41	31	36.5	37
Median NL	971	839	1033	1233
% of individuals with positive NL	76.7	70.1	75.5	74.3
Between-group statistics	–	OR (95% CI) = 0.834 (0.492–1.412), *p* = 0.498, BF_10_ = 0.154		
Adjusted R^2^	0.023			

N with positive NL	*N* = 79	*N* = 75	*N* = 83	*N* = 75
Median NL	2042	1627	2177	2089
Between-group statistics	–	B (95% CI) = 0.042(-0.359–0.442), *p* = 0.838, BF_10_ = 0.125		
Adjusted R^2^	0.006			


*NL – net loss, positive NL means the customer lost money during data collection period, sum of stakes is higher than sum of winnings. OR – odds ratio of having a positive net loss in the pooled prompted group compared to control group. B – estimated change in adjusted NL in the pooled prompted group compared to control group. Higher age was associated with higher proportion of individuals with positive net loss and with higher net loss among individuals with positive net loss. BF_*10*_ – Bayes factor of the alternative hypothesis against the null hypothesis. For the proportion of customers with positive NL: estimates for effect of age (OR (95% CI) = 1.021(1.005–1.036), *p* = 0.009), estimates for effect of gender (being a male, OR (95% CI) = 0.709(0.451–1.117), *p* = 0.138). For the size of positive NL: estimates for effect of age (B (95% CI) = 0.012(0.001–0.024), *p* = 0.035), estimates for effect of gender (being a male, B (95% CI) = -0.090(-0.439–0.260), *p* = 0.616).*

## Discussion

The prompt to set a voluntary, removable deposit limit of optional size did not appear to be effective in reducing gambling intensity in users of an online gambling platform with online slot-machines as the preferred gambling category. The findings hold true both on whole-group level and among the 10% most involved gamblers. Higher age was associated with higher gambling intensity, which could be explained by the increase of income with age. The findings correspond to the results of previous research providing no clear evidence for the effectiveness of pre-commitment tools (Ladouceur et al., 2012). The failure of this particular intervention to influence gambling intensity can be partially explained by the intervention’s non-intrusive character. The customers were only exposed to the limit-setting prompt once, setting the limit was voluntary with no upper amount limit, and it was relatively easy to increase or remove the limit. It makes sense to suggest that actually setting a reasonable limit and adhering to it does help to limit one’s gambling, and one could speculate that a tool’s failure to have any impact is due to a design flaw. One of the most crucial aspects of pre-commitment tools is that they not only have to be set but also adhered to Ladouceur et al. (2012). No between-group differences in gambling intensity were found despite the fact that the prompted groups had higher inclination to set a limit. This suggests that the customers in most need of a limit either choose not to set it, choose to increase/remove it or choose an ineffective limit size.

Prompted limit-setters who chose not to increase or remove their limit showed lowest gambling intensity in two intervention groups, which is in line with the suggestion that deposit limits can be effective when they are adhered to. However, this assumes that this group is not the one that would have lowest gambling intensity even without a prompt and that these customers do not simply abandon one gambling platform for another one. Moreover, prompting does seem to increase rates of limit setting (Table 1) and could be a part of design of future pre-commitment tools also targeting the setting of reasonable limits and adhering to the limits. Setting a deposit limit without a prompt was associated with higher gambling intensity, which corresponds to previous research (Nelson et al., 2008). The unprompted limit-setters may be the ones knowing they need help to control their spending. Increasing/removing a prompted or unprompted limit showed the same association and these associations could be used in identification of high-risk gamblers.

The highly skewed distribution of both time- and money-related variables with a very high density of observations to the left of the scale close to zero and a long thin tail to the right, suggests the existence of subgroups of gamblers—for example, ones who can be categorized as high- and low-intensity (Chamberlain et al., 2017). Possible subgroups are also implied by the quantile regression plot (Supplementary Figure 2) and distribution of active gambling days as well as last gambling days (Figure 3). Variations in gambling intensity on the whole-group level should not be considered an ultimate measure of gambling sustainability because there may be differences in what constitutes a meaningful change in the subgroups. The subgroups may be defined by subjecting the user activity time-series data to a classifying statistical analysis, for instance latent class analysis. Moreover, the character of and transitions between commonly used subgroups of gamblers, such as non-problem, at-risk, and problem gamblers, might be an area of interest when talking about meaningful changes in gambling intensity. Exploration of these aspects requires matching of customer behavior data from an online gambling platform with data on problem gambling severity and finding markers for the categories of interest in the behavioral data. Although the current study only used data on customer activity in the platform without a sophisticated subgroup analysis, the results provide an insight into the possible effects of a deposit-limit setting prompt on gambling intensity. Finding a substantial difference in the percentage of the highest-intensity gamblers would indicate that the current RG-tool has the potential to protect customers that are, arguably, in most need of an intervention. The absence of the between-group differences in the current trial does motivate further modification and evaluation of pre-commitment tools, such as using multiple prompts and creating a communication plan for gamblers who choose to increase or remove their limit.

One of the study’s limitations is the use of a non-intrusive one-time prompt, which may be insufficient for a substantial effect. The prompt did not include any comprehensive educational information on the importance to set a limit and did not enable the customer to make an informed choice on pre-commitment. The prompt can also be considered disproportionate to the 90-day tracking that followed the registration. Although the current trial answers the question whether prompting gamblers to set a deposit limit in this particular manner affects subsequent NL, it cannot answer another important question – that is whether setting a limit has an effect on gambling intensity. Another trial design, for instance involving randomization of the participants to set a mandatory limit of a certain size, is required in order to answer the latter question. The sample was partially self-selected as selection of slot-players occurred after the randomization, and randomization could potentially influence the customers’ inclination to play different types of games. However, the proportions of slot-players in the randomization groups did not differ. No information on the customers’ activity outside the particular gambling platform was obtained. Adjusting the monetary data due to confidentiality issues makes it impossible to anchor the gambling intensity described in the current study to other published data. The results do not generalize to gamblers with other preferred gambling category than online slots or to gamblers with multiple preferred gambling categories. NL as measure of gambling intensity is contaminated with promo-credits from the gambling operator and occasional wins. Future research should consider using deposits as a primary outcome measure, as they are likely to represent the own money that a gambler has decided to spend on gambling. Deposits do not take negative values making statistical analyses easier. Number of logins and number of deposits were suggested as secondary outcome measures. However, given the relevant measures already reported – such as number of active gambling days and sum of deposits – adding number of logins and number of deposits was considered to be redundant for the current study, and occasional between-group differences related to these variables would be hard to interpret given lack of differences based on variables presented in the current study.

As Finland holds one of Europe’s few gambling monopolies, a discussion of how this could affect the study’s sample would be in place. Despite monopolized gambling market, where the publicly run gambling company Veikkaus is the only one allowed to provide gambling services, gambling in Finland is both accessible and available (Castrén et al., 2018). All the common online gambling types are provided by Veikkaus with some restrictions related to monetary transactions: the highest amount of money allowed on a gambling account is 20 000 EUR, customers have to set a deposit limit of optional size when registering an account and no deposits are allowed between 24:00 and 06:00, also, high-speed games have a loss limits of 1000 EUR per day or 2000 EUR per month (Ministry of the Interior in Finland, 2018). Levels of gambling problems in Finland seem to be higher than in other Nordic (Salonen et al., 2018) and other European countries (Calado and Griffiths, 2016). The study was carried out using data from a different local gambling monopoly called Paf from Åland islands, a jurisdiction in Finland. Only customers from mainland Finland – not Åland islands – were analyzed in the current trial. The restrictions mentioned above do not apply to games provided by Paf. Paf is considered to be a non-monopoly company in Finland, and only 14.3% of Finnish population report ever having used a non-monopoly gambling service which is also associated with higher rates of gambling related harms (Castrén et al., 2018). This could suggest that the sample in the current study was more susceptible to experience gambling harms than a representative sample of Finnish gamblers. On the other hand, although Paf is a non-monopoly company, it is considered to be a regulated company that is allowed to provide gambling services in mainland Finland as an exception, which suggests small differences between the study sample and a representative sample of gamblers from Finland.

Pre-commitment is suggested as a mandatory responsible gambling measure in several jurisdictions (Australian Government, Productivity Commission, 2010; Statens Offentliga Utredningar [SOU], 2017). However, given the results reported in previous and current research, there is no evidence of pre-commitment tools being able to decrease gambling-related harm in online gambling platforms and this should be reflected in the official recommendations. Until appropriate evidence is found, marketing of certain common ways of implementing pre-commitment tools – voluntary, of optional size and relatively easy to increase/remove - as a strategy for reducing gambling-related harm in online settings should be considered problematic. All existing and upcoming pre-commitment designs should be studied thoroughly in order to ensure that they serve their purpose, and—most importantly—do no harm.

## Data Availability

The data analyzed in this study was obtained from Ålands Penningautomatförening (Paf), a publicly governed gambling company from the Åland Islands, Finland. The following restrictions apply: non-disclosure agreement between Stockholm University and Paf hindering Stockholm University from sharing the data sets with a third party. Requests to access these datasets should be directed to Paf, info@pafcasino.com.

## Author Contributions

EI was a project coordinator and had major responsibility for all the parts of the research process. EI and KM conducted the statistical analyses. All authors contributed to creating the manuscript and the design of the study.

## Conflict of Interest Statement

EI’s Ph.D. position is funded by a grant from Ålands Penningautomatförening (Paf), which is a publicly governed gambling operator from Finland. KM’s Ph.D. position is funded by a grant from Svenska spel’s independent research council. Svenska Spel is a publicly governed gambling operator from Sweden. PC has been the primary investigator of two larger treatment studies on pathological gambling funded by the Public Health Agency of Sweden. He has also received 3-year funding from FORTE, a government agency under the Swedish Ministry of Health and Social Affairs, for an Internet-delivered treatment for concerned significant others of people with problem gambling. In addition, he has received three research grants (Svenska spel and Paf) specifically devoted to only cover the university costs of employing two Ph.D.-students and one postdoc. Finally, PC has served as an unpaid gambling expert for the National Board of Health and Welfare (Socialstyrelsen) which is a government agency in Sweden under the Ministry of Health and Social Affairs.

## Footnotes

1 https://osf.io/g67gu/
