# Supplementary material for: Deposit Limit Prompt in Online Gambling for Reducing Gambling Intensity: A Randomized Controlled Trial
Source: Front Psychol. 2019 Mar 28;10:639. doi: 10.3389/fpsyg.2019.00639 (PMC6455077; doi:10.3389/fpsyg.2019.00639)
Supplement: Supplementary file 1 [file Table_1.DOCX]

Supplementary Material

**Effects of a Deposit Limit Prompt in an Online Gambling Platform for Reducing Gambling Intensity: A Randomized Controlled Trial**

Ekaterina Ivanova^*^, Kristoffer Magnusson, Per Carlbring

*** Correspondence:** Ekaterina Ivanova: ekaterina.ivanova@psychology.su.se

# Supplementary Figures and Tables

## Supplementary Figures

| 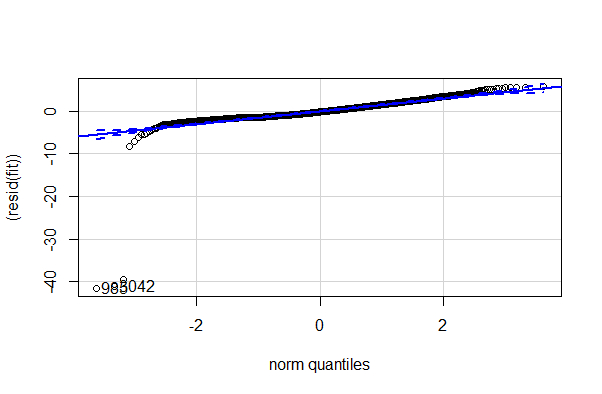 | 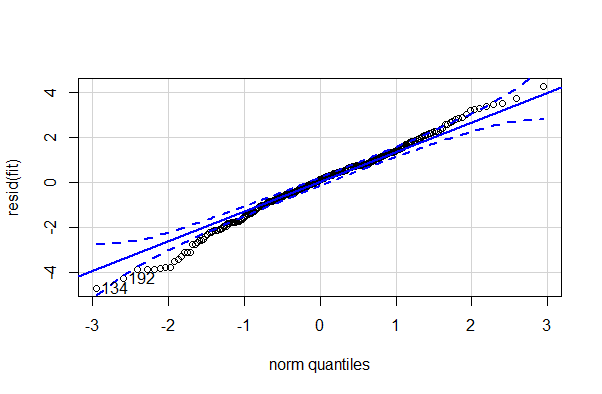 |
| --- | --- |
| **(A)** | **(B)** |
| 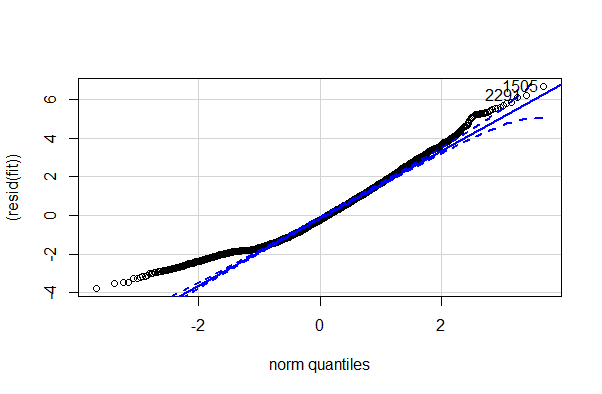 | 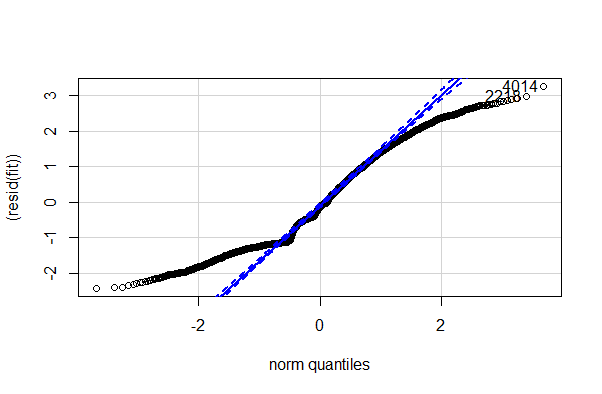 |
| **(C)** | **(D)** |
| **Supplementary Figure 1.** QQ-plot of residuals for linear regression on positive net loss for the whole sample **(A)** and for the subgroup of most involved gamblers **(B)**, on total sum of deposits **(C)** and total number of gambling days **(D)**. | |

| 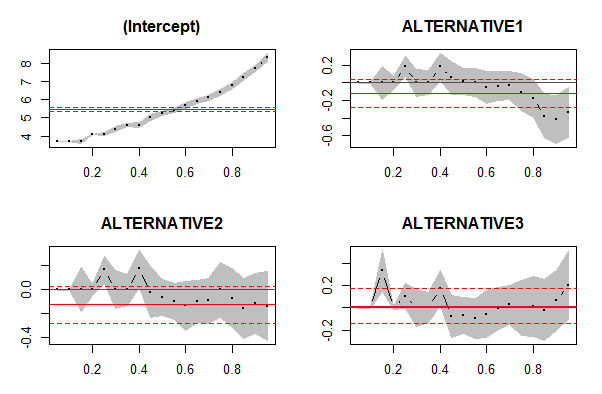 |
| --- |
| **Supplementary Figure 2**. Quantile regression of log-transformed NL for customers with positive NL. Intercept – control group, ALTERNATIVE1 – at-registration group, ALTERNATIVE2 – pre-deposit group, ALTERNATIVE3 – post-deposit group. |

## Supplementary Tables

| **Supplementary Table 1** | | | | |
| --- | --- | --- | --- | --- |
| Comparisons between the prompted groups and the control group regarding total sum of deposits and total number of gambling days. | | | | |
|  | Control | At-registration | Pre-deposit | Post-deposit |
| Sum of deposits |  |  |  |  |
| Median | 100 | 106 | 109 | 100 |
| N analysed^1^ | 1031 | 1048 | 1071 | 1010 |
| Between-group statistics | - | B (95% CI) =0.046(-0.068-0.160), p =.429, BF_10_ = 0.044 | | |
| Adjusted R^2^ |  | 0.058 | | |
| Total N of gambling days |  |  |  |  |
| Median | 3 | 3 | 3 | 3 |
| N analysed^2^ | 1063 | 1097 | 1105 | 1050 |
| Between-group statistics |  | B (95% CI) =0.041(-0.042-0.124), p =.329, BF_10_ = 0.050 | | |
| Adjusted R^2^ |  | 0.052 | | |
| ^1^ N=168 (N _at-registration/pre-deposit/post-deposit/control_ = 50/39/45/34, χ^2^ = 3.505, *p* = .320) never made a deposit only having played for promo-money and were excluded from the deposit analysis in order to log-transform the sum of deposit variable.  ^2^ N = 13 cancelled all their bets after having placed them (N _at-registration/pre-deposit/post-deposit/control_ = 1/5/5/2) and were removed from the analysis of total number of gambling days in order to log-transform the variable.  B – estimated change in adjusted sum of deposits and total number of gambling days in the pooled prompted group compared to control group.  BF_10_ - Bayes factor of the alternative hypothesis against the null hypothesis.  **For the sum of deposits:** estimates for effect of age (B (95% CI) =0.033(0.029-0.037), p <.001), estimates for effect of gender (being a male, B (95% CI) =0.206(0.101-0.311), p <.001)  **For the total number of gambling days:** estimates for effect of age (B (95% CI) =0.022(0.019-0.025), p <.001), estimates for effect of gender (being a male, B (95% CI) =-0.051(-0.127-0.026), p =.195) | | | | |
